# Ethanol and Cyanide: A Case Report on Toxic and Nutritional Optic Neuropathy Associated With Alcohol and Tobacco

**DOI:** 10.7759/cureus.97067

**Published:** 2025-11-17

**Authors:** Nicolas Nicolaou, Despina Nicolaou, Lisa Douglas, Savvas Christou

**Affiliations:** 1 General Surgery/Vascular Surgery, Addenbrooke's Hospital, Cambridge University Hospitals NHS Foundation Trust, Cambridge, GBR; 2 General Surgery/Ophthalmology, Queen Mary University of London, London, GBR; 3 Obstetrics and Gynaecology, Peterborough City Hospital, Peterborough, GBR; 4 Obstetrics and Gynaecology, Addenbrooke's Hospital, Cambridge University Hospitals NHS Foundation Trust, Cambridge, GBR; 5 General Medicine, Addenbrooke's Hospital, Cambridge University Hospitals NHS Foundation Trust, Cambridge, GBR; 6 Ophthalmology, St. George Hospital, Paphos, CYP

**Keywords:** alcohol-induced neurotoxicity, ethanol-related optic nerve damage, nutritional optic neuropathy, painless bilateral loss of vision, retinal nerve fiber layer (rnfl) thinning, vitamin b12 and thiamine deficiency

## Abstract

Tobacco-alcohol amblyopia, an acquired optic neuropathy, is now classified as toxic and nutritional optic neuropathy (TNON). A 40-year-old man presented with gradual, painless, symmetrical visual loss and central scotomas. Pupillary reactions were normal, with no relative afferent pupillary defect (RAPD), though colour vision was markedly reduced. Optic discs appeared normal with early temporal pallor in the left eye. Optical coherence tomography (OCT) revealed thinning of the peripapillary retinal nerve fibre layer (pRNFL) in the temporal quadrant and decreased macular thickness. The patient reported heavy alcohol consumption for five years and smoking two packs of cigarettes daily. Laboratory findings indicated vitamin B12 deficiency and low folate levels. Despite supplementation, visual recovery was limited due to longstanding retinal nerve fibre loss. Chronic alcoholism is indirectly toxic to the optic nerve, as vitamin B12 and folate deficiencies impair mitochondrial function. This leads to the selective injury of unmyelinated ganglion cell axons, particularly within the papillomacular bundle. Optic discs initially appear normal, but progressive thinning of the pRNFL results in temporal pallor later extending to all quadrants. The parafoveal ganglion cell-inner plexiform layer (GCL+IPL) and macular thickness may be reduced. Smoking further exacerbates nutritional deficiencies; however, cyanide in tobacco is also directly toxic to the optic nerve. Diagnosis relies on careful history-taking, the evaluation of macular and pRNFL thickness, visual field testing, and colour vision assessment. This case highlights the importance of early recognition of TNON, particularly when optic discs appear normal, to enable timely nutritional therapy and visual recovery.

## Introduction

Tobacco-alcohol amblyopia was first described during the Second World War and the Cuban epidemic [1]. Alcohol was once thought to exert a direct toxic effect on the optic nerve, similar to other neurotoxins. However, it is now recognised that alcohol-related optic neuropathy is secondary to vitamin B12, folate, and thiamine deficiencies, as visual function often improves with nutritional replacement. Tobacco contains cyanide, which can directly damage the optic nerve and worsen nutritional deficiency [1-3]. Because many individuals with chronic alcoholism also smoke, the combined toxic and nutritional effects can result in toxic and nutritional (deficiency) optic neuropathy (TNON) [2,4]. It typically presents with painless, bilateral, and symmetrical visual loss, often accompanied by red-green dyschromatopsia and central or cecocentral scotomas [5,6].

The underlying mechanism is thought to involve mitochondrial dysfunction selectively affecting ganglion cell fibres of the papillomacular bundle (PMB) [5,6]. Evidence from Leber hereditary optic neuropathy (LHON) supports this, as mitochondrial DNA mutations in LHON cause similar ganglion cell damage and clinical presentation to TNON [1].

Optic atrophy may not be evident in early stages, delaying recognition. Nerve fibre damage may begin in less apparent regions such as the optic tracts and chiasm, later involving ganglion cell axons within the retinal nerve fibre layer (RNFL) of the PMB [1,5-9]. Optical coherence tomography (OCT) demonstrates the thinning of the peripapillary RNFL (pRNFL) and macular ganglion cell layers, enabling earlier diagnosis [10-13]. Contrast sensitivity may be reduced, and visual field analysis may reveal central or centrocecal scotomas. In later stages, temporal pallor of the optic discs becomes more pronounced [10-13]. We present a case illustrating this condition and discuss its clinical features and pathophysiology to promote earlier recognition and intervention.

## Case presentation

A 40-year-old man presented with gradual, progressive, bilateral, painless visual loss, worsening over recent weeks. He stopped driving due to blurred central vision and safety concerns. He denied headaches, diplopia, or recent systemic illness. The patient reported daily alcohol use for five years since the COVID-19 pandemic, averaging 3-5 units of vodka (40% alcohol) per day, and smoked two packs of cigarettes daily. There was no history of illicit drug use or regular medication. Family and personal histories were negative for glaucoma or other ocular disease. 

**Figure 1:**
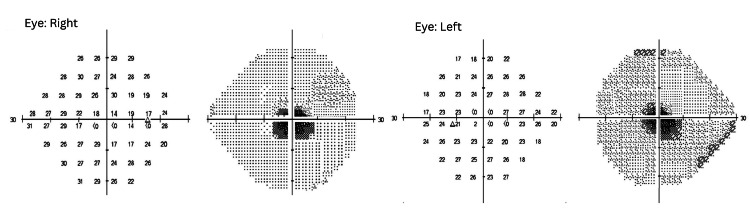
Humphrey Visual Field (24-2) analysis of the right and left eyes Visual fields demonstrating bilateral central scotomas with preserved peripheral fields. Reliability regarding the size and density of the scotomas was limited due to suboptimal patient concentration; however, the results were consistent with a bilateral reduction in visual acuity and subsequent findings.

On examination, best-corrected visual acuity (BCVA) was right eye (OD) −0.25/−0.25 × 180, 6/36−2, and left eye (OS) −0.25/−0.50 × 170, 6/36. Near vision was N18 in both eyes, with no improvement on pinhole. Near vision was N18 in both eyes, with no improvement on pinhole. Colour vision testing with Ishihara plates was markedly reduced bilaterally (1/14). Pupillary responses were normal and symmetrical, with no relative afferent pupillary defect (RAPD). Ocular motility was full in both eyes. Confrontation visual field testing revealed bilateral central scotomas with preserved peripheral fields, confirmed on Humphrey Visual Field (HVF) testing (Figure 1). 

**Figure 2:**
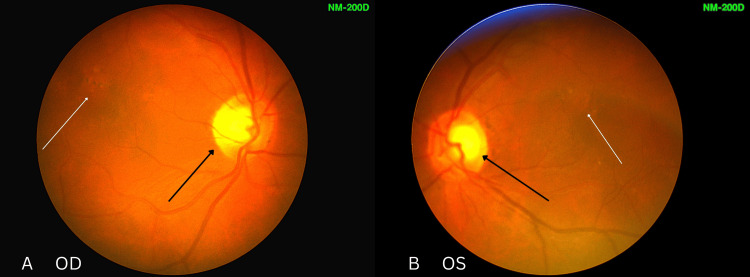
Fundus photographs of the (A) right eye and (B) left eye Fundus images showing normal optic discs with mild temporal pallor, more evident in the left eye. Pigmentary macular changes in both eyes initially obscured the diagnosis of optic neuropathy. OD: right eye; OS: left eye

**Figure 3:**
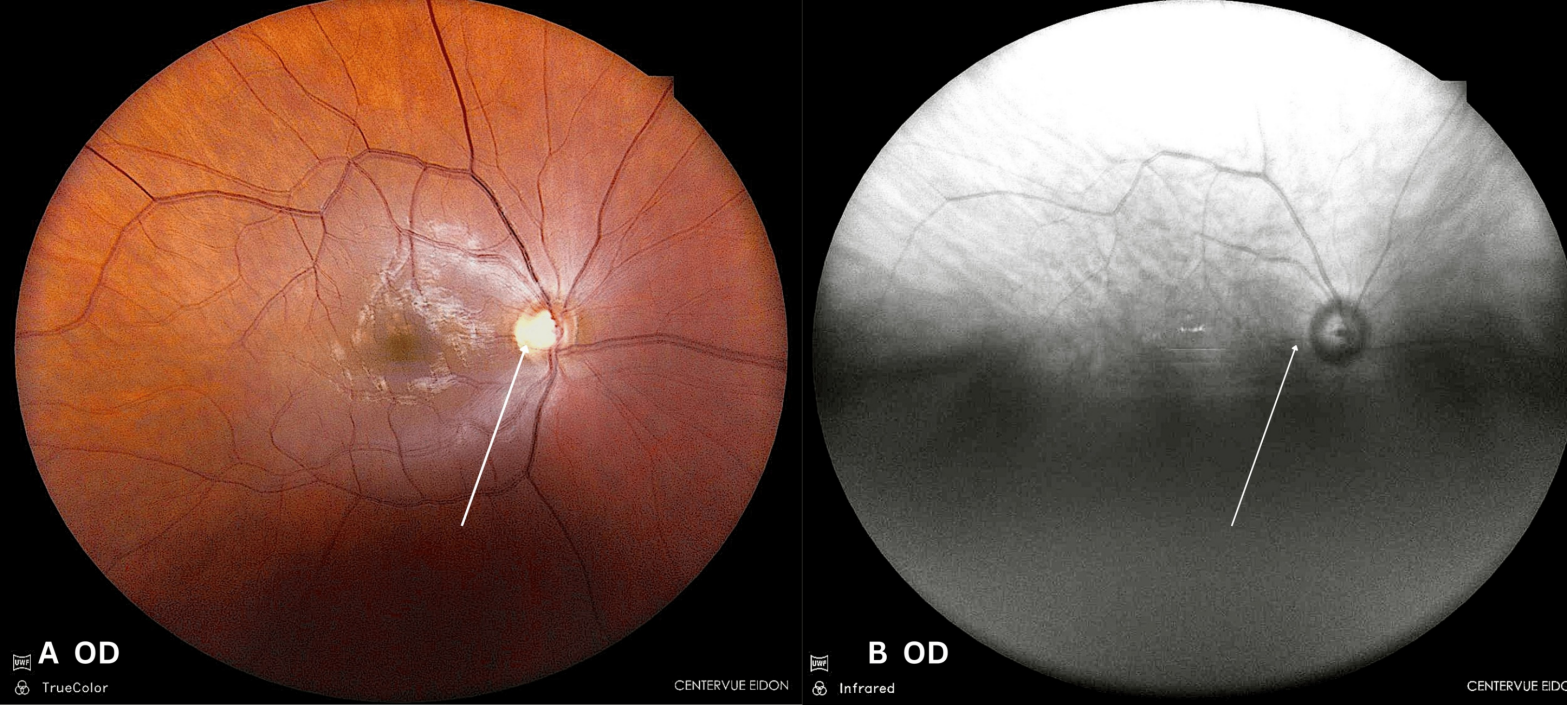
(A) True-colour and (B) infrared fundus imaging of the right eye True-colour and B infrared fundus images of the right eye showing an optic disc that appears normal, with early temporal pallor not readily apparent. OD: right eye

**Figure 4:**
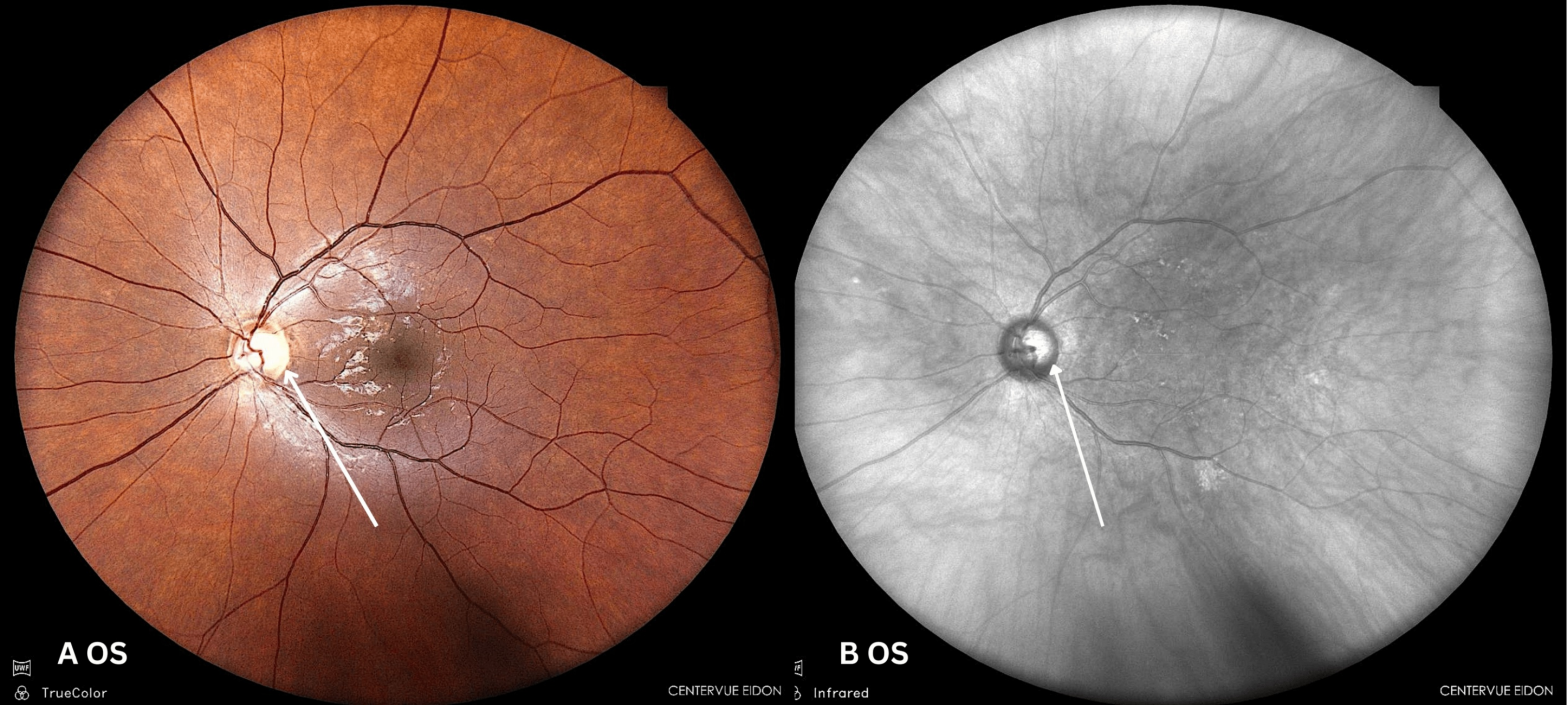
(A) True-colour and (B) infrared fundus imaging of the left eye True-colour and B infrared fundus images of the left eye demonstrating mild temporal pallor of the optic disc. OS: left eye

**Figure 5:**
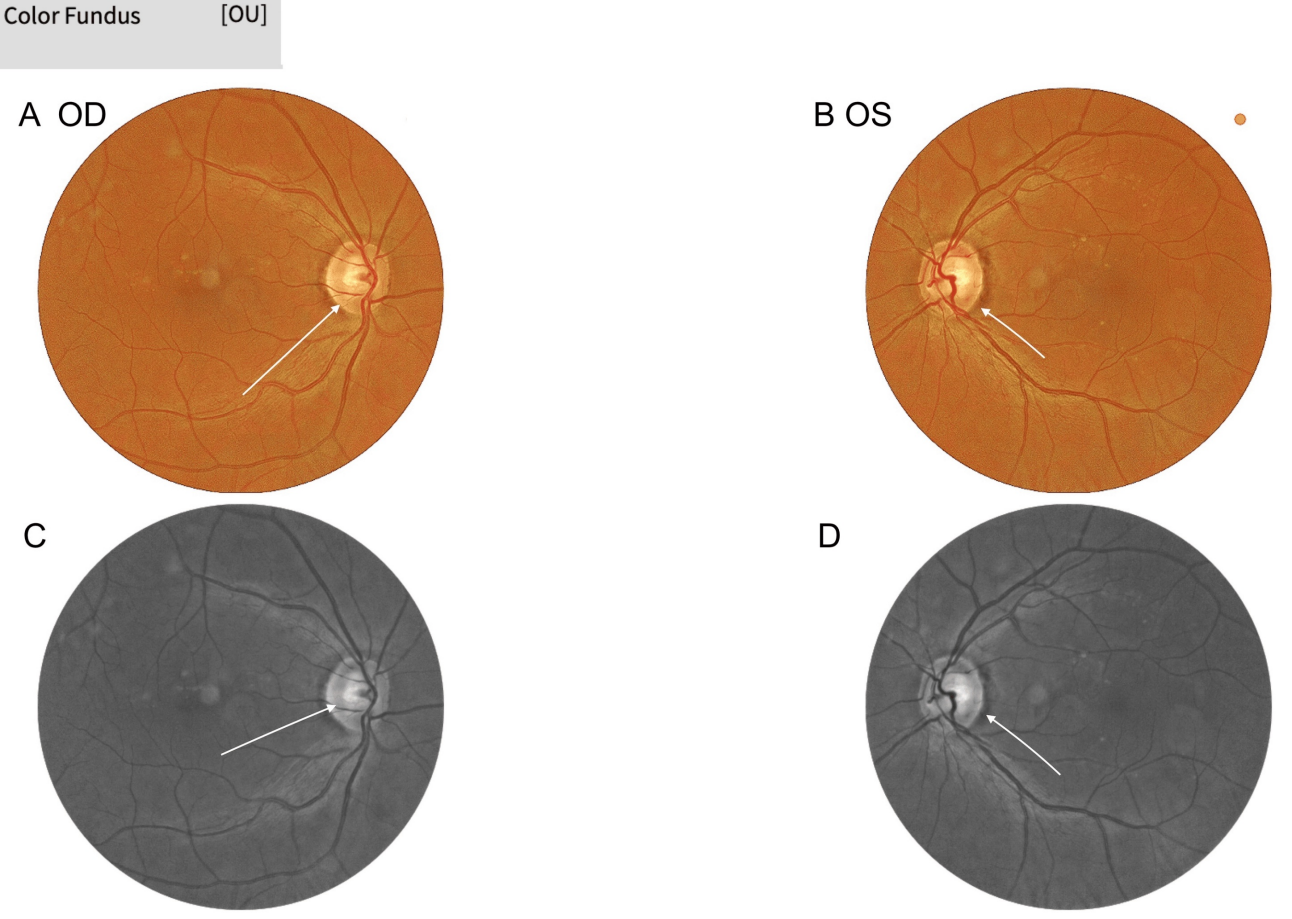
Colour and grayscale fundus photographs of both eyes (A, B) Colour fundus images of the right and left eyes showing early temporal disc pallor. (C, B) Grayscale fundus images confirming temporal pallor, more pronounced in the left eye. OD: right eye; OS: left eye

Anterior segment examination was normal. Intraocular pressures were 15 mmHg (OD) and 14 mmHg (OS). Direct fundoscopy showed mild left temporal pallor, with cup-to-disc ratios of 0.8 (OD) and 0.6 (OS). Macular pigmentary changes in both eyes made the diagnosis of optic neuropathy challenging. No disc oedema or retinal haemorrhages were seen (Figures 2-5).

**Figure 6:**
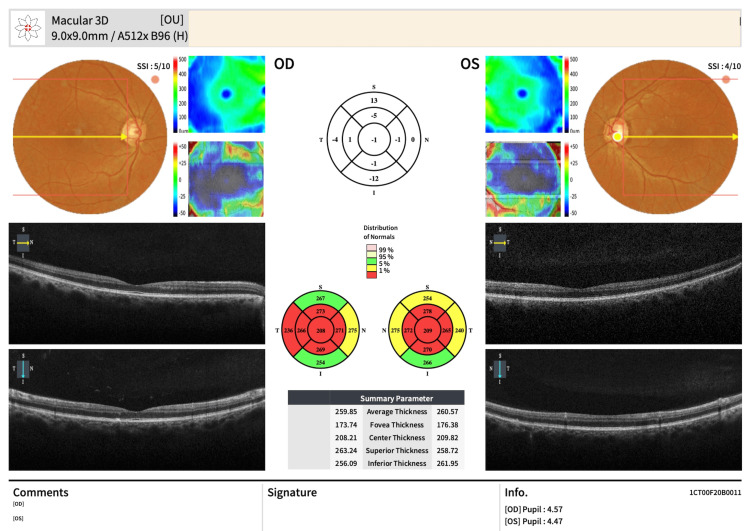
Spectral-domain OCT macular cube comparison of macular thickness values with normative data The ETDRS grid from the macular cube scan assesses retinal thickness across macular regions. In this case, red sectors indicate thickness measurements below the first percentile of the normative database in the right eye and left eye, consistent with reduced macular thickness in nutritional optic neuropathy. OCT: optical coherence tomography; ETDRS: Early Treatment Diabetic Retinopathy Study; OD: right eye; OS: left eye

Spectral-domain OCT of the macula using the Early Treatment Diabetic Retinopathy Study (ETDRS) grid showed that multiple macular sectors, particularly the central and temporal regions, were below the first percentile compared with age-matched normative values (Figure 6).

**Figure 7:**
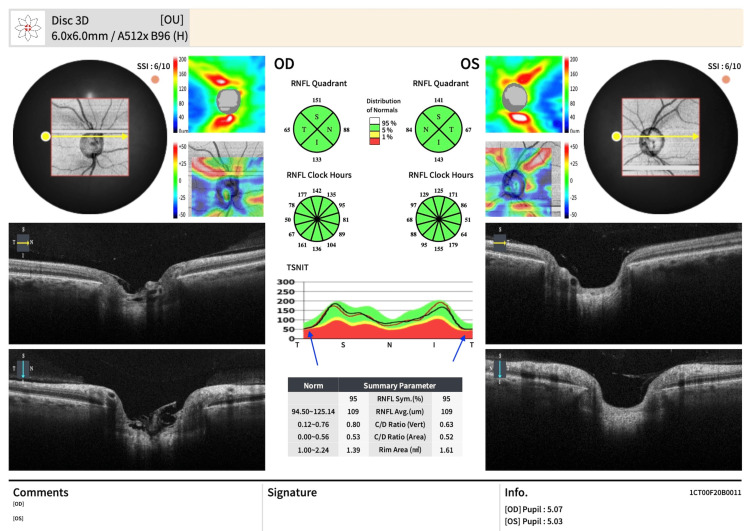
3D OCT scan of both optic nerves The TSNIT profile demonstrates bilateral flattening of the temporal curve, indicating temporal pRNFL thinning. The vertical cup-to-disc ratio in the right eye was 0.80, above the normal range, suggesting optic nerve damage. OCT: optical coherence tomography; TSNIT: The temporal-superior-nasal-inferior-temporal; pRNFL: peripapillary retinal nerve fibre layer; OD: right eye; OS: left eye; OU: both eyes

Peripapillary OCT analysis revealed thinning of the pRNFL in the temporal quadrant (65 µm in the right eye and 67 µm in the left eye). The temporal-superior-nasal-inferior-temporal (TSNIT) plot of the RNFL demonstrated flattening of the temporal curve in both eyes (Figure 7). 

Laboratory tests (Table 1) showed low serum vitamin B12 (153 pg/mL) and folate (3.39 ng/mL). Liver function tests revealed elevated gamma-glutamyl transferase (GGT), consistent with alcoholic liver disease. A diagnosis of TNON was made based on the clinical presentation and findings. The patient was referred to an alcohol cessation clinic and commenced on intramuscular vitamin B12, oral folate, and thiamine. However, there was minimal improvement after four weeks, with BCVA of 6/24 (OD) and 6/24−2 (OS).

**Table 1:** Laboratory results Laboratory results demonstrated vitamin B12 deficiency (153 pg/mL) and low folate levels (3.39 ng/mL), with elevated GGT, ALT, and AST. A diagnosis of TNON and alcoholic liver disease was made based on the clinical presentation and findings. GGT: gamma-glutamyl transferase; ALT: alanine aminotransferase; AST: aspartate aminotransferase; ALP: alkaline phosphatase; TNON: toxic and nutritional optic neuropathy

Parameter	Result	Reference range	Interpretation
Vitamin B12	153 pg/mL	200-900 pg/mL	Low
Folate	3.39 ng/mL	≥3 ng/mL	Low-normal
GGT	182 U/L	0-55 U/L	Elevated
ALT	78 U/L	0-40 U/L	Mildly elevated
AST	92 U/L	0-40 U/L	Elevated
ALP	106 U/L	44-147 U/L	Normal
Bilirubin (total)	0.8 mg/dL	0.1-1.2 mg/dL	Normal

## Discussion

Progressive, painless, bilateral, and symmetrical visual loss warrants a detailed clinical history, as TNON is often under-recognised, particularly when fundoscopic findings appear normal [3,6,7]. Early recognition reduces the risk of permanent RNFL loss and improves the likelihood of visual recovery [4]. Over time, the optic disc may become hyperaemic, with temporal pallor later as observed in our patient. Dyschromatopsia presents early, and a blue-yellow colour defect may be observed preceding a red-green defect in both eyes [5]. A monocular defect may suggest optic neuritis, a demyelinating process, or a compressive lesion. Neuroimaging should be performed to exclude any tumour or other pathology of the optic nerves in cases of visual loss [2,3]. In the early stages, reduced contrast sensitivity may be detected [7]. 

Pupillary reactions are normal or sluggish. RAPD is absent when both optic nerves are symmetrically affected but may be present if one eye is more severely involved. Individuals with alcoholism often have limited insight into their condition and typically present with central or cecocentral scotomas, with preserved peripheral visual fields, significantly impairing daily functioning [5-7]. Colour perception across all hues is affected with central scotomas as reflected by the markedly reduced Ishihara score of 1/14 in this patient.

Cecocentral scotomas result from injury to the unmyelinated ganglion cell fibres of the PMB [5]. These small-calibre axons are particularly vulnerable to metabolic and mitochondrial dysfunction compared with other optic nerve fibres [5]. Evidence from LHON, a mitochondrial genetic disorder that causes painless, progressive central vision loss, supports the role of mitochondrial dysfunction as a key pathogenic mechanism underlying nutritional optic neuropathy (NON) [1]. 

NON, driven by chronic alcohol use, may lead to deficiencies of cyanocobalamin (vitamin B12), folate, and thiamine due to poor dietary intake, malabsorption, and metabolic depletion. Other factors, such as prolonged proton pump inhibitor (PPI) use, bariatric surgery, and a vegan diet, can also contribute to these deficiencies. The mechanisms of axonal damage remain unclear, but vitamin B12 deficiency, in particular, leads to the accumulation of formic acid, which disrupts the electron transport chain, impairs mitochondrial function, and reduces adenosine triphosphate (ATP) synthesis [4]. This results in injury to parvocellular retinal ganglion cell axons in the PMB, which are crucial for central vision and colour perception, leading to dyschromatopsia [8,9].

Evidence supporting vitamin deficiencies includes reports of visual improvement following nutritional supplementation, although recovery is typically limited to the early stages [4-6]. In one case involving 20 years of daily omeprazole (PPI) use, NON was associated with vitamin B12 deficiency. After the cessation of alcohol and omeprazole and the initiation of vitamin B12 supplementation, improvements were observed in visual acuity, colour vision, and resolution of the central scotoma [4]. Our patient showed minimal visual recovery despite adequate supplementation, likely due to irreversible nerve fibre damage. 

Tobacco smoking compounds NON through the toxic effects of cyanide, a known neurotoxin. Chronic exposure to cyanide and free radicals in tobacco smoke directly damages the highly metabolic "type-P" ganglion cell axons in the PMB. This occurs through the disruption of the mitochondrial respiratory cycle, reduced ATP production, increased oxidative stress, and subsequent apoptosis [7]. Nicotine also contributes to indirect ischemic optic nerve injury through vasoconstriction, reducing blood flow and resulting in secondary ischemic damage [7,8].

The effects of tobacco and alcohol in TNON can be seen in the analysis of the RNFL. In a case-control study involving 100 patients with chronic alcoholism and 100 healthy controls, RNFL thinning was observed in 50% of alcohol cases. Among the cases, nine were tobacco-only users, 29 alcohol-only, and 62 combined users. RNFL thinning was observed in all quadrants among cases compared with controls. Within the alcohol-only group, greater RNFL thinning was noted in those with a greater Alcohol Use Disorders Identification Test (AUDIT) score above 20, although this difference was not statistically significant. Among individuals who only smoked (with no alcohol use), those with a higher Fagerström Nicotine Dependence score (>5) had thinner RNFLs than non-smokers [10]. This thinning was statistically significant in the superior, inferior, and temporal quadrants, suggesting that tobacco has a greater negative effect on RNFL thickness than alcohol alone, though their combined effect is more pronounced [10-12].

A systematic review demonstrated significant thinning of the pRNFL and the macular ganglion cell-inner plexiform layer (GCL+IPL) within the parafoveal region, along with a reduction in average macular thickness in individuals with alcohol use disorder (AUD) [11-14]. In contrast, the central fovea, which lacks ganglion cells, showed no statistically significant reduction in thickness [13]. These findings suggest selective damage to retinal ganglion cell axons, particularly those within the PMB. The authors concluded that the quantitative assessment of the pRNFL and macular GCL+IPL may serve as a valuable biomarker in the evaluation of TNON [11-14].

In our patient, OCT macular cube analysis revealed reduced macular thickness, with the ETDRS grid (highlighted in red in Figure 6) indicating values thinner than 99% of the normative database in both eyes. These findings correlate with the bilateral central scotomas and reduced visual acuity. pRNFL thinning was also observed in the temporal quadrant (Figure 7). Table 2 summarises the clinical features of TNON, and Table 3 outlines the differential diagnoses. 

**Table 2:** Symptoms, signs, and diagnostic tests for nutritional optic neuropathy TNON requires a detailed history and multiple investigations to assist in its accurate diagnosis, as the optic discs may initially appear normal. *Spectral-domain OCT (e.g., Carl Zeiss Meditec) can also assess GCL+IPL thickness. RAPD: relative afferent pupillary defect; pRNFL: peripapillary retinal nerve fibre layer; RNFL: retinal nerve fibre layer; GCL+IPL: ganglion cell-inner plexiform layer; VF: visual field; OCT: optical coherence tomography; MRI: magnetic resonance imaging; TNON: toxic and nutritional optic neuropathy Table created by the authors based on [1-14].

Symptoms	
Painless, progressive, bilateral, and symmetrical visual loss	
Central or cecocentral scotoma	
Dyschromatopsia	
Loss of contrast sensitivity	
Signs	
RAPD is absent if symmetrical [1-5]	
Normal or hyperaemic optic disc (early stages). Temporal and then diffuse optic disc pallor (late stage) [1-5]	
Thinning of the pRNFL in the papillomacular bundle (early stage). Thinning of the RNFL involving all quadrants (late stage) [11-13]	
Thinning of macular GCL+IPL and average macular thickness [11-13]	
Test	Finding/purpose
Colour vision tests (Ishihara plates)	Dyschromatopsia
VF (Humphrey Visual Field Analysis)	Central or cecocentral scotoma
RNFL OCT	To monitor pRNFL thinning and macular thickness and GCL+IPL*
MRI	To exclude compressive or demyelinating disease
Laboratory analysis (vitamin B12, folate, thiamine)	To confirm or exclude nutritional deficiency as the underlying cause

**Table 3:** Differential diagnoses of TNON highlighting key distinguishing clinical and investigative features LHON: Leber hereditary optic neuropathy; DOA: dominant optic atrophy; MS: multiple sclerosis; MRI: magnetic resonance imaging; NMOSD: neuromyelitis optica spectrum disorder; NAION: non-arteritic anterior ischaemic optic neuropathy; AAION: arteritic anterior ischaemic optic neuropathy; GCA: giant cell arteritis; ESR: erythrocyte sedimentation rate; CRP: C-reactive protein; TNON: toxic and nutritional optic neuropathy Table created by the authors based on [1-7].

Category	Condition	Key distinguishing features
Hereditary optic neuropathies	LHON	Mitochondrial disorder; young males; maternal inheritance pattern; central vision loss; family history; no nutritional deficiency
	DOA	Autosomal dominant inheritance; gradual bilateral central vision loss; onset in childhood or early adulthood; temporal disc pallor
Demyelinating/inflammatory	Optic neuritis (MS-related)	Unilateral, painful visual loss; reduced colour vision; MRI shows demyelinating lesions; visual recovery common after corticosteroids
	NMOSD	Bilateral or recurrent optic neuritis; poor recovery; positive aquaporin-4 antibodies; associated transverse myelitis
Compressive/infiltrative	Optic nerve or chiasmal compression (e.g., pituitary adenoma, meningioma)	Gradual visual loss; bitemporal hemianopia; MRI shows a compressive lesion; disc pallor without systemic nutritional cause
Ischaemic	NAION	Sudden, painless monocular loss; segmental optic disc oedema; altitudinal visual field defect; vascular risk factors
	AAION, GCA	Elderly patients; sudden severe visual loss; systemic symptoms (jaw claudication, scalp tenderness); raised ESR/CRP
Toxic	Ethambutol, linezolid, methanol, amiodarone, isoniazid	Bilateral, symmetrical visual loss; clear history of drug/toxin exposure; reversibility depends on duration and dose
Nutritional deficiencies	Vitamin B12, folate, thiamine, or copper deficiency	Associated systemic features (anaemia, neuropathy, glossitis); history of malnutrition, vegan diet, or gastrointestinal surgery
Other inflammatory/infectious	Sarcoidosis, tuberculosis, syphilis, lymphoma	Optic nerve or chiasmal involvement with systemic signs; abnormal MRI; positive serology or biopsy confirmation

The mainstay of treatment is vitamin supplementation with folic acid and oral vitamin B12, together with intramuscular hydroxocobalamin (vitamin B12) therapy. Management also includes discontinuation of alcohol and tobacco use, along with rehabilitation. Our patient showed minimal improvement due to extensive retinal nerve fibre loss. 

## Conclusions

TNON requires a detailed history of nutrition, alcohol intake, and tobacco use, as optic discs may appear normal early on. Visual loss with central or cecocentral scotomas is typically bilateral, symmetrical, progressive, and painless. Deficiencies in vitamin B12 and folate are linked to mitochondrial dysfunction, which selectively affects small-calibre axons of the PMB. Tobacco smoke compounds this damage through direct toxic effects on the highly metabolic "type-P" ganglion cells. OCT assessment of the pRNFL, macular thickness, and macular GCL+IPL provides valuable biomarkers for TNON. Up to 50% of individuals with TNON show RNFL thinning. In later stages, temporal pallor and optic atrophy become evident across all quadrants of the optic nerve. Early recognition and prompt vitamin B12 and folic acid supplementation, along with cessation of alcohol and tobacco use, can improve or stabilise visual function.
